# The Role of Conjunctival Microvasculation Combined with Echocardiography in Evaluating Pulmonary Arterial Hypertension in Systemic Lupus Erythematosus

**DOI:** 10.1155/2021/2135942

**Published:** 2021-11-26

**Authors:** Jiangbiao Xiong, Shujiao Yu, Ren Liu, Xia Fang, Rui Wu

**Affiliations:** The First Affiliated Hospital of Nanchang University, Nanchang 330006, China

## Abstract

**Objective:**

To explore the role of conjunctival microvasculation combined with echocardiography in evaluating the prognosis of pulmonary arterial hypertension in systemic lupus erythematosus (SLE-PAH).

**Methods:**

We prospectively compared the conjunctival microvascular changes in 17 SLE-PAH patients and 34 SLE patients without PAH in our hospital from January 2020 to December 2020, and we observed the characteristics of conjunctival microvascular changes in SLE-PAH patients. We analyzed the correlation between the corresponding conjunctival microvascular changes and cardiopulmonary function and evaluated the predictive value of the vessel density (VD) and the microvascular flow index (MFI) of conjunctival microvasculation combined with echocardiography in SLE-PAH.

**Results:**

Compared with SLE patients without PAH, the ischemic areas in conjunctival microvasculation were significantly increased in SLE-PAH patients. The VD and MFI of conjunctival microvasculation are significantly correlated with N-terminal prohormone of brain natriuretic peptide and 6-minute walking distance. Combined with the VD and MFI, it can improve the accuracy of echocardiography in assessing the risk of death due to SLE-PAH (94.1% vs. 82.2%).

**Conclusion:**

The ischemic area, VD, and MFI of conjunctival microvasculation in SLE-PAH patients can indicate the occurrence of severe SLE-PAH and improve the accuracy of echocardiography in evaluating the prognosis of SLE-PAH.

## 1. Introduction

Systemic lupus erythematosus (SLE) is an autoimmune disease involving multiple organs. Pulmonary arterial hypertension (PAH), one of its serious complications, is difficult to treat and is one of the causes of death due to SLE [1, 2]. Right heart catheterization is the gold standard for the clinical diagnosis and assessment of pulmonary hypertension, but due to its invasiveness and high cost, it is not suitable as a mean of regular evaluation during treatment. Echocardiography is recommended for its simplicity for the indirect diagnosis and dynamic assessment of pulmonary hypertension [3, 4]. However, the consistency of the echocardiography and right heart catheterization results is still controversial [5]. Therefore, looking for more markers to improve the accuracy of the clinical assessment of pulmonary hypertension, early identification of high-risk patients, and timely intervention are essential to improve the prognosis of SLE-PAH patients. A previous study confirmed that conjunctival microvasculation can effectively assess the hypercoagulable or prethrombotic state of SLE [6]. Thus, this study observed the characteristics of conjunctival microvasculation in SLE-PAH patients and evaluated its value combined with echocardiography in the assessment of SLE-PAH.

## 2. Materials and Methods

### 2.1. Subjects

Seventeen consecutive SLE-PAH patients who were admitted to the Department of Rheumatology and Immunology of the First Affiliated Hospital of Nanchang University from January 2020 to December 2020 and a control group of 34 SLE patients without PAH who were hospitalized during the same period were selected based on the matched course of the disease, age, and sex. All patients were evaluated based on the 2019 European League Against Rheumatism/American College of Rheumatology Classification Criteria for Systemic Lupus Erythematosus [7]. Patients with pulmonary hypertension were divided into three groups: low, medium, and high risk according to the 2015 European Guidelines for the diagnosis and risk assessment of pulmonary hypertension [8]. Clinical evaluations, including pulmonary hypertension functional classification, 6-minute walking distance (6MWD), serum uric acid (SUA), N-terminal prohormone of brain natriuretic peptide (NT-proBNP), and red blood cell volume distribution width (RDW), were performed.

### 2.2. Conjunctival Microvasculation

An SLM-7E digital slit lamp was used to detect conjunctival microvasculation. Each subject rotated their eyeballs up, down, left, and right to fully expose the required observation range. Eight conjunctival microvasculation images were observed and recorded when the binoculars were in four different positions. The observations included the following: ischemic area: more than 3 capillary grid areas without vessels under a 40x microscope; reticulum deformity: the capillaries increase, the mesh decreases, and the dendrites become grid-like; microangioma can be divided into local round, fusiform, cystic dilation, or isolated and scattered around the vessel; and wound spot: vascular blind end-shaped brown, purple, or dark blue material deposition [6]. Bleeding is exudative (fuzzy vessel wall), and there are ruptures (spots, patches) around capillaries. For vessel density (VD) calculation method, the image under a 40x mirror was divided into 16 grids with three horizontal lines and three vertical lines, and the number of lines passing through the grid was calculated and averaged. For microvascular flow index (MFI), the image under a 100x microscope was divided into four directions, and the average was taken after integration. Normal flow was counted as 3 points, sluggish flow was 2 points, intermittent flow was 1 point, and no flow for at least 20 seconds was 0 points [9].

### 2.3. Echocardiography

A color Doppler ultrasound with a probe frequency of 1.7 ~ 3.4 MHz was used to detect right atrial pressure (RAP), systolic pulmonary artery pressure (sPAP), and tricuspid annular plane systolic excursion (TAPSE) [10].

### 2.4. Statistical Analysis

SPSS 22.0 was used for data analysis. The *t*-test was used to compare the two sample means conforming to the normal distribution; otherwise, the Mann–Whitney test was used. The chi-square test was used for comparisons between enumeration dates. The Kruskal–Wallis test was used for multigroup comparisons of ranked data. Spearman's test was used for the correlation analysis, and multiple discriminant analysis was used to compare the accuracy of the predictive risk assessment. The difference was statistically significant at *P* < 0.05.

## 3. Results

### 3.1. Comparison of the Characteristics between SLE Patients with and without PAH

Comparison of the clinical data showed that Raynaud's phenomenon, pericardial effusion, and positive rates of antiphospholipid antibodies in SLE-PAH patients were significantly higher than those in SLE patients without PAH. Conjunctival microvasculation in all patients with SLE had various manifestations, such as twisting, dilation, ischemia, hemorrhage, reticular malformation, and wound spots. Among them, conjunctival vasodilation, vascular distortion, and injury points were the most common, followed by ischemic areas, reticulum deformity, hemorrhage, and microangioma. The incidence of SLE-PAH ischemic areas was significantly higher than that of SLE patients without PAH (*P* < 0.05) (Table 1).

### 3.2. Correlation between VD and MFI of Conjunctival Microvasculation and SLE-PAH-Related Parameters

**Figure 1:**
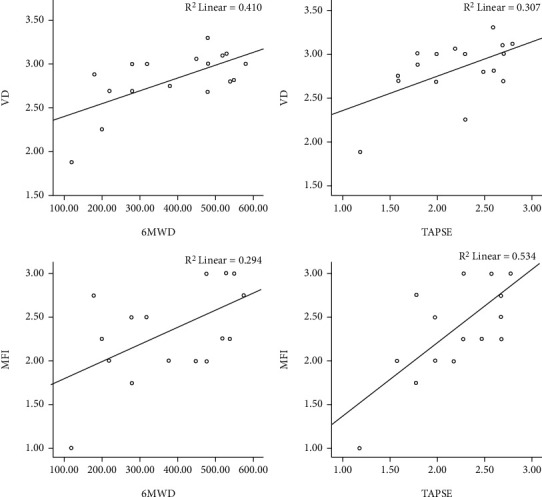
Scatter plot of the correlation analysis of VD, MFI, 6MWD, and TAPSE.

Spearman correlation analysis indicated that VD was significantly negatively correlated with NT-proBNP, UA, and RAP; meanwhile, MFI was significantly negatively correlated with NT-proBNP, sPAP, and RAP. In addition, VD and MFI were positively correlated with 6MWD and TAPSE, respectively (Table 2, Figure 1).

### 3.3. Comparison of the Parameters between Different Risk Groups and Discriminant Analysis

**Figure 2:**
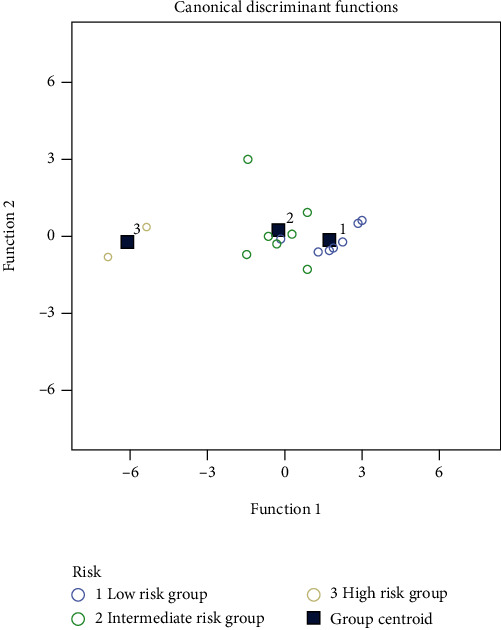
Discriminant analysis of the echocardiography combined with VD and MFI in three groups of patients with different risk stratifications.

According to the risk assessment in pulmonary arterial hypertension in the 2015 ESC/ERS guidelines for the diagnosis and treatment of pulmonary hypertension, SLE-PAH patients were divided into low risk, intermediate risk, and high risk groups. The results suggested that TAPSE, NT-proBNP, VD, and MFI were significantly different in the three risk groups, but there was no significant difference in terms of sPAP, RDW, RAP, and SUA. Multivariate discriminant analysis revealed that the accuracy of the echocardiographic parameters, PAH, RAP, and TAPSE for PAH risk assessment was 82.4%, and the accuracy after combining VD and MFI could be increased to 94.1% (Table 3, Figure 2).

## 4. Discussion

Systemic lupus erythematosus is an autoimmune disease that often occurs in women of childbearing age. Studies have shown that SLE-PAH is one of the most important causes of death in SLE patients following neuropsychiatric lupus and lupus nephritis. In particular, severe PAH has a very poor prognosis, and most of the late deaths are due to progressive heart and lung failure [11]. Early recognition and treatment of pulmonary hypertension, regular assessment and treatment of the standard, and control of pulmonary hypertension to a low-risk state are currently important treatment strategies for pulmonary hypertension [12–14]. Effective and accurate assessment of pulmonary hypertension is an indispensable part in the standard treatment [15].

Microcirculation refers to the blood circulation between arterioles and venules. It is the peripheral part of the circulatory system and an important place for the exchange of substances between blood and tissue cells. The conjunctiva is a common site for detecting systemic microcirculation, which is less affected by external temperature, is closer to the changes in visceral microcirculation, and can better reflect diseases of the visceral vessels [16]. Pulmonary hypertension can cause increased right heart load, reduced pulmonary flow, and mismatched ventilation and perfusion, resulting in tissue ischemia and hypoxia, and affect peripheral microcirculation through oxidative stress and vasoconstriction [17]. The local microvessels of the conjunctiva are significantly reduced, and the capillaries are closed, showing a state of disconnection, disappearance, local paleness, and even the appearance of ischemic areas, which are the manifestations of severe microcirculation disorders. This study showed that 6 (35.3%) patients in the SLE-PAH group had ischemic areas, all of whom had moderate to severe pulmonary hypertension. The incidence of ischemia was significantly higher than that of lupus patients without PAH (35.3% versus 14.7%, *P* = 0.042), which suggested that the ischemic areas were features of conjunctival microvasculation in SLE patients with severe pulmonary hypertension. By observing the VD and MFI of conjunctival microvasculation through a slit lamp at a magnification of 40-100 times, we found that the VD and MFI were significantly correlated with NT-proBNP and 6MWD (*P* < 0.05), suggesting that the changes in VD and MFI might be related to decreased cardiopulmonary function. Importantly, this study also found that the VD and MFI of conjunctival microvasculation could improve the accuracy of echocardiographic-related parameters in assessing the risk of pulmonary hypertension.

Invasive hemodynamic monitoring of pulmonary hypertension has certain risks, high technical requirements, and high costs, and it is not suitable for long-term repeated use [18]. The ultrasound parameters of the risk assessment in pulmonary arterial hypertension in the 2015 ESC/ERS guidelines included only RAP and the right atrium area [8]. However, this study found that compared with RAP, TAPSE showed more significant changes in different risk groups, which suggested that it might have more advantages in the risk assessment of SLE-PAH [19–21].

The study has several limitations. First, when conjunctival microcirculation is detected, involuntary movements of the eyeball will cause instability in image acquisition and affect the accuracy of the data. Second, the sample size of SLE-PAH patients was not large enough. In the future, a larger sample will be needed for the verification of these findings.

## 5. Conclusion

This study proposes a new method for evaluating the prognosis of SLE-PAH. The ischemic area, VD, and MFI of conjunctival microcirculation combined with echocardiography can improve the accuracy of risk assessment of PAH in SLE.

## Figures and Tables

**Table 1:** Comparisons of the characteristics between SLE-PAH patients and SLE patients without PAH.

Characteristics	SLE-PAH (*n* = 17)	SLE without PAH (*n* = 34)	*P* value
Female sex, *n* (%)	15 (88%)	31 (91%)	0.625
Age, years ± SD	44.4 ± 14.6	41.7 ± 12.9	0.751
Pleural effusion, *n* (%)	2 (6.7%)	7 (4.7%)	0.646
Raynaud's phenomenon, *n* (%)	17 (56.7%)	48 (32.0%)	0.010^∗^
Oral ulcer, *n* (%)	4 (13.3%)	26 (17.3%)	0.592
Alopecia, *n* (%)	8 (26.7%)	66 (44.0%)	0.078
Rash, *n* (%)	9 (30.0%)	50 (33.3%)	0.723
Pericardial effusion, *n* (%)	6 (20.0%)	2 (1.3%)	≈0.00^∗^
Lupus nephritis, *n* (%)	5 (16.7%)	30 (20.0%)	0.674
Cytopenia, *n* (%)	3 (10.0%)	6 (4.0%)	0.169
Elevated ESR, *n* (%)	19 (63.3%)	80 (53.3%)	0.315
Low C3, *n* (%)	26 (86.7%)	114 (76.0%)	0.200
Low C4, *n* (%)	23 (76.7%)	103 (68.7%)	0.383
Anti-dsDNA antibody, *n* (%)	18 (60.0%)	62 (41.3%)	0.060
LA, *n* (%)	24 (80.0%)	66 (44.0%)	≈0.00^∗^
ACA, *n* (%)	17 (56.7%)	13 (8.7%)	≈0.00^∗^
Conjunctival microvasculation			
Ischemic areas, *n* (%)	6 (35.3%)	5 (14.7%)	0.042^∗^
Reticulum deformity, *n* (%)	4 (23.5%)	13 (38.2)	0.233
Microangioma, *n* (%)	8 (47.1%)	7 (41.2)	0.101
Twisting, *n* (%)	16 (94.1%)	27 (79.4)	0.242
Dilation, *n* (%)	8 (47.1%)	24 (70.6)	0.131
Wound spot, *n* (%)	13 (76.5)	17 (50)	0.081
Hemorrhage, *n* (%)	5 (29.4%)	6 (17.6)	0.472

SD: standard deviation; ESR: erythrocyte sedimentation rate; LA: lupus anticoagulant; ACA: anticardiolipin antibodies. ^∗^*P* < 0.05.

**Table 2:** Correlation between VD and MFI of conjunctival microvasculation and SLE-PAH-related parameters.

			sPAP	NT-proBNP	6MWD	SUA	RDW	RAP	TAPSE
Spearman's rho	VD	Correlation coefficient	-0.441	-0.528^∗^	0.574^∗^	-0.560^∗^	-0.323	-0.637^∗∗^	0.539^∗^
		Sig. (2-tailed)	0.076	0.029	0.016	0.019	0.306	0.006	0.026
	MFI	Correlation coefficient	-0.537^∗^	-0.546^∗^	0.504^∗^	-0.350	-0.305	-0.689^∗∗^	0.683^∗∗^
		Sig. (2-tailed)	0.026	0.023	0.039	0.168	0.335	0.002	0.003

VD: vessel density; MFI: microvascular flow index; sPAP: systolic pulmonary artery pressure; NT-proBNP: N-terminal prohormone of brain natriuretic peptide; 6MWD: 6-minute walking distance; SUA: serum uric acid; RDW: red blood cell volume distribution width; RAP: right atrial pressure; TAPSE: tricuspid annular plane systolic excursion. ^∗^*P* < 0.05, ^∗∗^*P* < 0.01.

**Table 3:** Comparison of the parameters between different risk groups.

Parameters	Low risk (*n* = 7)	Intermediate risk (*n* = 7)	High risk (*n* = 3)	*P* value^△^
sPAP (mmHg)	32.0 ± 8.0	59.8 ± 31.2	61.5 ± 9.2	0.23
TAPSE (cm)	2.7 ± 0.1	2.1 ± 0.4	1.4 ± 0.3	0.012^∗^
RAP (mmHg)	5.8 ± 1.5	8.2 ± 3.7	15 ± 0	0.147
NT-proBNP (pg/mL)	247.8 ± 250.1	1456.5 ± 719.8	5418.5 ± 2617	0.003^∗^
SUA (ummol/L)	430.8 ± 117.9	436.7 ± 92.3	518.5 ± 89.8	0.67
RDW (%)	15.4 ± 2.3	21.7 ± 10.2	18.5 ± 1.7	0.315
VD (n/mm^2^)	2.7 ± 0.3	2.3 ± 0.2	1.7 ± 0.4	0.006^∗^
MFI	2.8 ± 0.4	2.3 ± 0.3	1.5 ± 0.7	0.019^∗^
Conjunctival microvascular changes	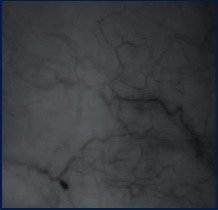	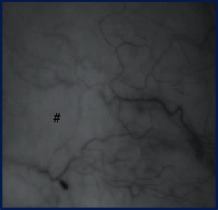	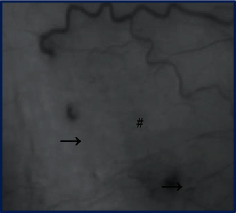	

sPAP: systolic pulmonary artery pressure; TAPSE: tricuspid annular plane systolic excursion; RAP: right atrial pressure; NT-proBNP: N-terminal prohormone of brain natriuretic peptide; SUA: serum uric acid; RDW: red blood cell volume distribution width; VD: vessel density; MFI: microvascular flow index. ^∗^*P* < 0.05. △ Kruscal-Wallis test; # ischemic areas; ⟶ microangioma.

## Data Availability

The data used to support the findings of this study will be available from the corresponding author upon reasonable request.
